# Transcriptional regulation and ubiquitination-dependent regulation of HnRNPK oncogenic function in prostate tumorigenesis

**DOI:** 10.1186/s12935-021-02331-x

**Published:** 2021-12-02

**Authors:** Huan-Lei Wu, Sen-Mao Li, Yao-chen Huang, Qi-Dong Xia, Peng Zhou, Xian-Miao Li, Xiao Yu, Shao-Gang Wang, Zhang-Qun Ye, Jia Hu

**Affiliations:** 1 Department of Geriatrics, Tongji Hospital, Tongji Medical College, Huazhong University of Science and Technology, Wuhan, 430030 China; 2 Department of Urology, Institute of Urology, Tongji Hospital, Tongji Medical College, Huazhong University of Science and Technology, Liberalization Ave, No. 1095, Wuhan, 430030 P.R. China; 3 Department of Urology, Peking University First Hospital, Peking University, BeijingBeijing, 100034 China

**Keywords:** HnRNPK, Prostate cancer, miRNA, Post-transcriptional regulation, Degradation

## Abstract

**Background:**

Heterogeneous nuclear ribonucleoprotein K (HnRNPK) is a nucleic acid-binding protein that regulates diverse biological events. Pathologically, HnRNPK proteins are frequently overexpressed and clinically correlated with poor prognosis in various types of human cancers and are therefore pursued as attractive therapeutic targets for select patients. However, both the transcriptional regulation and degradation of HnRNPK in prostate cancer remain poorly understood.

**Methods:**

qRT-PCR was used to detect the expression of HnRNPK mRNA and miRNA; Immunoblots and immunohistochemical assays were used to determine the levels of HnRNPK and other proteins. Flow cytometry was used to investigate cell cycle stage. MTS and clonogenic assays were used to investigate cell proliferation. Immunoprecipitation was used to analyse the interaction between SPOP and HnRNPK. A prostate carcinoma xenograft mouse model was used to detect the in vivo effects of HnRNPK and miRNA.

**Results:**

In the present study, we noted that HnRNPK emerged as an important player in the carcinogenesis process of prostate cancer. miR-206 and miR-613 suppressed HnRNPK expression by targeting its 3’-UTR in PrCa cell lines in which HnRNPK is overexpressed. To explore the potential biological function, proliferation and colony formation of PrCa cells in vitro and tumor growth in vivo were also dramatically suppressed upon reintroduction of miR-206/miR**-**613. We have further provided evidence that Cullin 3 SPOP is a novel upstream E3 ubiquitin ligase complex that governs HnRNPK protein stability and oncogenic functions by promoting the degradation of HnRNPK in polyubiquitination-dependent proteolysis in the prostate cancer setting. Moreover, prostate cancer-associated SPOP mutants fail to interact with and promote the destruction of HnRNPK proteins.

**Conclusion:**

Our findings reveal new posttranscriptional and posttranslational modification mechanisms of HnRNPK regulation via miR-206/miR**-**613 and SPOP, respectively. More importantly, given the critical oncogenic role of HnRNPK and the high frequency of SPOP mutations in prostate cancer, our results provide a molecular rationale for the clinical investigation of novel strategies to combat prostate cancer based on SPOP genetic status.

**Supplementary Information:**

The online version contains supplementary material available at 10.1186/s12935-021-02331-x.

## Background

Prostate cancer (PrCa) is one of the most prevalent malignancies among males in the Western world and represents considerable health concerns in many industrialized countries [1]. Nearly one-third of patients with Gleason scores ≥ 7 and higher will experience biochemical recurrence and the emergence of advanced-stage disease, particularly metastatic progression after radical prostatectomy. The disease further progresses to castration-resistant prostate cancer (CRPC) approximately 12–24 months after androgen deprivation therapy, which still lacks an effective cure, and patients rapidly progress to incurable stage PrCa with a mean survival time of only 16–18 months [2, 3]. Therefore, there is a need to more accurately understand the mechanism of oncogenesis and to develop additional effective therapeutics targeting pivotal oncogenes, cancer-related signal transduction pathways, and epigenetic regulators [4].

HnRNPK is specific for HnRNP family members, and it can participate in numerous cellular processes in the nucleus and cytoplasm. In addition to having the same functions as other HnRNPs, it can regulate DNA transcription, pre-mRNA processing and translation, particularly with regard to the process of oncogene expression [5, 6]. These features all make HnRNPK exhibit multiple roles in the cell cycle, apoptosis and tumor metastasis [7]. According to previous results, there is a close association between tumors and HnRNPK; it often shows high expression in a variety of tumors and is closely associated with poor cancer prognosis in patients, including lung cancer, colorectal cancer, bladder cancer, hepatocellular carcinoma and neuroblastoma [7–11]. Ciarlo et al. [12] demonstrated that HnRNPK was strongly overexpressed in primary human PrCa and played a central role in neuroendocrine differentiation regulation. In addition, Wang et al. [13] showed that a novel transcriptional repressor complex containing Pur α and HnRNPK binds to the androgen receptor (AR) gene both in cell lines and in primary human prostate tissues. More recently, evidence has been provided for a role played by HnRNPK in the regulation of AR expression via a posttranscriptional mechanism [14, 15]. HnRNPK is a critical regulator of malignancy in the PrCa setting, and further understanding of its role in transcriptional regulation and degradation is required to treat PrCa.

The deregulation of small ncRNAs, such as microRNAs (miRNAs), has been recognized as one mechanism involved in the induction and progression of PrCa. It acts either as a tumor suppressor or promoter depending on the specific targets and tumor microenvironment [16, 17]. In addition, the ubiquitin–proteasome system governs a variety of biological processes and disease conditions, such as cell-cycle progression and malignant transformation. Notably, systematic sequencing studies revealed that recurrent somatic mutation is a key feature of PrCa and the most frequently mutated gene is SPOP (speckle-type POZ protein), which encodes a Cullin 3-based E3 ubiquitin ligase, with recurrent mutation in 10–15% of primary human PrCa [18]. SPOP has been shown to participate in diverse cellular processes and plays tumor suppressive and oncogenic roles in PrCa by targeting different substrates for ubiqutination-mediated proteolysis, including AR [19], steroid receptor coactivator 3 (SRC-3) [20], DEK, TRIM24 [21], BRD4 [22] and Nanog [23]. Furthermore, PrCa-associated SPOP mutants have been reported to be defective in binding with and promoting the proteasomal degradation of substrates leading to increased PrCa cell proliferation and invasion, indicating the loss-of-function of SPOP mutations and the tumor suppressive role of SPOP in PrCa [19, 22]. Therefore, identification of additional SPOP substrates would benefit PrCa clinical diagnosis and therapy.In this study, we identified that HnRNPK dysregulation in prostate carcinogenesis is correlated with a decrease in miR-206 and miR-613 expression. Furthermore, we found that miR-206 and miR-613 inhibit HnRNPK expression by directly targeting its 3’-UTR and thereby repress PrCa cell proliferation. Moreover, we explored the oncoprotein HnRNPK as a novel ubiquitin substrate of SPOP in the PrCa setting, and PrCa-associated SPOP mutants failed to promote the degradation of HnRNPK proteins.

## Materials and methods

### Patients and tissue samples

PrCa samples and adjacent normal tissue samples were collected during radical prostatectomy from PrCa patients between 2010 and 2016 at the Tongji Hospital, Tongji Medical College, Huazhong University of Science and Technology in Wuhan, China. The PrCa cases selected were based on a clear pathological diagnosis, follow-up data, and absence of androgen deprivation therapy, chemotherapy, radiotherapy or other anticancer treatment before surgery. All specimens had confirmed pathological diagnosis and were classified according to the WHO criteria. The clinicopathological patient information was collected and summarized in Additional file 1: Table S1. All protocols were approved by the Ethics Committee of Tongji Hospital, Tongji Medical College, Huazhong University of Science and Technology, and informed consent was obtained from all patients before surgery. All in vivo protocols were approved by the Institutional Animal Care and Use Committee of Tongji Hospital, Tongji Medical College, Huazhong University of Science and Technology.

### Bioinformatics analysis databases

The correlation of HnRNPK and SPOP expression with the biochemical recurrence of tumor patients were analyzed via Gene Expression Omnibus (https://www.ncbi.nlm.nih.gov/geo/). The miRNA target predicting algorithm TargetScan Release 7.1 (http://www.targetscan.org/vert_71/) was used to predict miRNAs targeting HnRNPK and their binding regions.

### Cell culture

HEK293, HEK293T and mouse embryonic fibroblasts (MEFs) cells were maintained in Dulbecco's Modified Eagle Medium (DMEM) (Life Technologies, CA) containing 10% fetal bovine serum (FBS) (Gibico), 100 units of penicillin and 100 mg/ml streptomycin. PrCa cell line PC-3, DU145, 22Rv1, C42, VCaP and LNCaP were obtained from ATCC and maintained in RPMI1640 (Life Technologies, CA) supplemented with 10% FBS. The normal prostate epithelial cells RWPE-1 (ATCC) were maintained in Keratinocyte-SFM (Gibco, GrandIsland, NY, USA). SPOP knockout and counterpart mouse embryonic fibroblasts (MEFs) were kindly offered by Dr. Wenyi Wei (Harvard Medical School, Boston, MA, USA) and Dr. Xiangpeng Dai (Jilin University, Changchun, China) No mycoplasma contamination was observed in these cell lines. All cells were cultured in a humidified atmosphere of 5% CO2 maintained at 37 °C.

### Oligonucleotide, plasmids construction

All small RNA molecules were ordered from RiboBio Co., Ltd. (Guangzhou, China), including miR-206 mimics, miR-613 mimics, mimics negative controls (mimics-NC), miR-206 inhibitor, miR-613 mimics, inhibitor negative controls (inhibitor-NC), miRNA mimics are double-stranded RNA molecules containing the miR-206 and miR-613 sequence, while miR-206 and miR-613 inhibitors are single stranded RNA molecules containing the miR-206 and miR-613 reverse complement sequence, which can competitively bind to endogenous miR-206 and miR-613. Flag-tagged HnRNPK, KEAP1, KLHL1, PLZF and KLHL20 were purchased from Addgene. Myc-tagged Cullins, pLenti-HA-SPOP WT, Flag-tagged SPOP WT, HA-tagged SPOP-WT or deletion of MATH domain/BTB domain-SPOP constructs and His-tagged Ub as gift from Dr. Wenyi Wei (Harvard Medical School, Boston, MA, USA). HA-HnRNPK and Various SPOP mutants were generated in this study.

### shRNAs and establishment of stable cell lines

The specific shRNAs vectors (Additional file 2: Table S2) to deplete endogenous HnRNPK (shHnRNPK: TRCN0000295992), SPOP (shSPOP: TRCN0000139181, TRCN0000144406) and CULLIN3 (shCULLIN3: TRCN0000307983, TRCN0000073346) were purchased from Sigma-Aldrich (St louis, MO, USA). The above shRNAs were cloned into the lentiviral vector pLKO.1. pLKO.1-puro eGFP shRNA(SHC005, Sigma-Aldrich) plasmid as the shRNA control. The HEK293T cells were transfected with above pLKO.1 carrying the specific sequences, along with the packaging plasmids, psPAX2 and pMD2.G. The virus particles were generated and collected 48 h, 72 h post-transfection, and then filtered with 0.45 μm filters (Millipore) and freshly used to infect cancer cells overnight in the presence of 4 μg/ml Polybrene (Sigma–Aldrich). After infection the PrCa cell lines, the cells were selected with 1 μg/ml puromycin (Sigma–Aldrich) for 72 h to eliminate the uninfected cells before harvesting the whole cell lysates for the subsequent biochemical assays. Knockdown efficiency was confirmed at both mRNA and protein levels.

### Antibodies and reagents

All antibodies were used at a 1:1000 dilution in 5% non-fat milk for Immunoblot. Anti-HnRNPK antibody (A1701) and Anti-SRC(A0324) antibody were purchased from Abclonal, respectively. Anti-GAPDH (ab37168) antibody, anti-P27 (ab32034) antibody, anti-CyclinD1 (ab134175) antibody, anti-SPOP((ab192233), anti-CDK6 (ab124821) antibody and anti-Bax (ab32503) antibody were purchased from Abcam. Anti-SRC3 (2126), polyclonal anti-Myc-Tag antibody (2278) and monoclonal anti-Myc-Tag (2276) antibodies were purchased from Cell Signaling. Polyclonal anti-Flag antibody (F-2425), monoclonal anti-Flag antibody (F-3165, clone M2), anti-Vinculin antibody (V-4505), anti-Flag agarose beads (A-2220), anti-HA agarose beads (A-2095), peroxidase-conjugated anti-mouse secondary antibody (A-4416) and peroxidase-conjugated anti-rabbit secondary antibody (A-4914) were purchased from Sigma. Monoclonal anti-HA antibody (MMS-101P) was purchased from Biolegend. Anti-GFP (8371-2) antibody was purchased from Clontech. Anti- NF-κB p65 (#8242) antibody, anti-E-cadherin (#3195) antibody, anti-Snail (#3879) antibody(CST), anti-β-Catenin (#8580) antibody, anti-BCL-2 (#15,071) antibody, anti-c-JUN (##9165) antibody, anti-c-MYC (#18,583) antibody and anti-CDK4 (#12,790) antibody were purchased from Cell Signaling Technology. Anti-Trim24 (sc-271266), anti-AR (N-20), anti-TRIM24/TIF1α (C-4), polyclonal anti-HA (SC-805), anti-p27 (SC-528) and anti-Nrf2 (sc-365949) were purchased from Santa Cruz Biotechnology. MG132 and cycloheximide (CHX) were purchased from Sigma–Aldrich.

### Cell transfection, lentivirus production

Cells were plated in growth medium at a density of 45 to 70%. The transfection was carried out using Lipofectamine RNAiMax (Invitrogen, Carlsbad, CA, USA) 24 h later according to the manufacturer's protocol. The final concentration of RNAs was 75 nM for each well. Others the transient transfections were performed using Lipofectamine 3000 (Invitrogen, Carlsbad, CA, USA) according to the manufacturer's protocol. For lentiviral-mediated overexpression of miR-206 and miR-613, the miR-206 and miR-613 sequence (pri miR-206 and pri miR-613) was cloned into H1-miRNA-CMV-GFP from GENECHEM, to generate the Lenti-miR-206 or Lenti-miR-613 construct. miR-NC (TTCTCCGAACGTGTCACGT) was cloned into the same backbone and the resulting construct Lenti-miR-NC served as a negative control. The transfection or infection efficiencies were detected by RT-qPCR.

### RNA isolation and quantitative real-time PCR

Total RNA of cells was extracted with TRIzol reagent (Invitrogen, Carlsbad, CA) according to the manufacturer’s protocol. Reverse transcription of microRNA and mRNA were done using RevertAid™ First Strand cDNA Synthesis Kit (Fermentas, Vilnius, Lithuania) and miProfile™ miRNA qPCR Primer (GeneCopoeia, Guangzhou,China). RT-qPCR analysis of miRNA was performed with the Platinum SYBR Green qPCR Supermix UDG kit (Invitrogen, Carlsbad, CA) using synthesized primers from GeneCopoeia (Guangzhou, China). The U6 primers were obtained from GeneCopoeia. All experiments were done in triplicate. The expression level values were normalized to those of the small nuclear RNA U6 as a control. Several primer sequences used are listed in Additional file 2: Table S2.

### Immunoblots and immunoprecipitation

Harvested cells washed by PBS and lysed in EBC buffer (50 mM Tris pH 7.5, 120 mM NaCl, 0.5% NP-40) supplemented with protease inhibitors (Complete Mini, Roche) and phosphatase inhibitors (phosphatase inhibitor cocktail set I and II, Calbiochem). The protein concentrations of lysates were measured by the Beckman Coulter DU-800 spectrophotometer using the BioRad protein assay reagent. Same amount of protein samples were separated by electrophoresis in sodium dodecyl sulfonate (SDS)-polyacrylamide gel and transferred onto a nitrocellulose filter (NC) membrane (Amersham). The membrane was incubated in 5% nonfat dry milk/TBST for 1 h; and then incubated with the primary antibody at 4 °C overnight. The membrane was washed with TBST for three times, followed by incubated with second antibody for 1 h at room temperature. Proteins of interest were measured by electrochemiluminescence (ECL) assay. For immunoprecipitation, Cells were washed with PBS and lysed in the IP lysis buffer (25 mM Tris•HCL pH7.4, 150 mM NaCl, 1 mM EDTA, 1% NP-40, 5% glycerol, 1 × Thermo protease inhibitor). Protein incubated 1000 μg of cell lysate with the primary antibody-conjugated beads at 4 °C for 4 h. The immunocomplexes were washed 3 times with IP lysis buffer before being resolved by SDS-PAGE and immunoblotted with the indicated antibodies.

### Protein half-life assays

Cells were treated with indicated condition. For half-life studies, 100 μg/ml cycloheximide (CHX, Sigma–Aldrich) was added to the cells after 36 h of post transfection. At the indicated time points, cells were harvested and protein concentrations were measured. Total 30 μg of the indicated whole cell lysates were separated by SDS-PAGE and protein levels were measured by immunoblot analysis.

### In vivo* ubiquitination assays*

For the In vivo ubiquitination assay, 293 T cells were seeded and transiently transfected with plasmids for His-Ub and other indicated proteins. Thirty-six hours after transfection, cells were treated with MG132 (20 μM) for 6 h before they were harvested. Cells were washed with PBS and lysed with IP lysis buffer (25 mM Tris·HCL pH7.4, 150 mM NaCl, 1 mM EDTA, 1% NP-40, 5% glycerol, 1 × Thermo protease inhibitor). 1000 μg of cell lysate were incubated with indicated antibody at 4 °C for 4 h and then incubated with Protein A/G plus agarose overnight. Beads were washed with lysis buffer for 3 times and detected by SDS-PAGE.

### Colony formation and MTS cell proliferation assay

The colony formation assay was conducted as previously described [24]. Briefly, exponentially growing cells were plated at approximately 2000 cells per well in 6-well plates after transfection. Culture medium was changed every 3 days. Colony formation was analyzed 12 days following infection by staining cells with 0.05% crystal violet solution for 30 min. The number of colonies was counted using an inverted microscope (Olympus, Japan). Cell proliferation was assessed by using the CellTiter 96 Aqueous One Solution Cell Proliferation. Assay kit (Promega, Madison, WI, USA) as previously described. Briefly, RNA transfected cells were grown in 96-well plates at a density of 2000 cells/well. Cell growth was measured daily for 4 days. At each time point, 20 µl of CellTiter 96 Aqueous One Solution was added and incubated. Absorbance was detected by a microplate reader (Bio-Rad, Berkeley, CA, USA) at 490 nm.

### Cell cycle

At 72 h after transfection, cells were fixed in 70% cold ethanol, incubated with RNase A (Sigma,St. Louis, MO, USA) and stained by propidium iodide (PI) (Nanjing KeyGen Biotech Co., Ltd., Nanjing, China) staining solution. After staining, the cells were analyzed on a FACSort flow cytometer (BD Biosciences, San Diego, CA, USA). The data were processed by CELL quest software (BD Biosciences).

### Luciferase reporter assay

HnRNPK 3’UTR reporter and control constructs were purchased from GENECHEM. Tumor cells overexpressing miR-206/613 and miR-NC cultured in 48-well plates were co-transfected with 1.5 mg of firefly luciferase reporter and 0.35 ng Renilla luciferase reporter with Lipofectamine RNAiMax (Invitrogen, Carlsbad, CA, USA). 24 h post transfection, firefly luciferase activities were measured using the Dual Luciferase Assay (Promega) and the results were normalized with Renilla luciferase according to the manufacturer’s protocol.

### Immunohistochemistry (IHC)

Formalin-fixed, paraffin-embedded tissue sections (5 µm) were deparaffinized in xylene and rehydrated with gradient concentrations of ethanol. The tissue sections were stained with specific antibodies against HnRNPK (1:400) (Abcam, ab32969). Sections incubated with secondary antibodies in the absence of primary antibodies were used as negative control. Hematoxylin was used for counterstaining. Slides were viewed and photographed under a light microscope.

### Xenograft model of PrCa in nude mice

For mouse xenograft assay, six-week-old BALB/c-nu/nu mice were randomly divided into two or three different experimental groups. Prostate cancer cells (1 × 10^6^) were collected and suspended in 100 μl PBS mixing with Matrigel (BD 356,234, 2:1) and injected into the nude mouse. Tumor onset was measured with calipers at the site of injection every 3–4 days by two trained laboratory staff members at different times on the same day, starting 12 days after injection when appreciable tumor formed subcutaneously. Tumor volume was determined by measuring the length (L) and width (W) with a Vernier caliper every four days and applying the formula, V = 0.5 × (L × W^2^), where L is the longest diameter and W is the shortest diameter. Animals were euthanized and xenografts were harvested 40 days after injection and tumors were weighed, and target gene expression were evaluated. Nude mice were manipulated and cared for according to NIH Animal Care and Use Committee guidelines in the Experiment Animal Center of the Tongji Medical College of Huazhong University of Science and Technology, China.

### Statistical analysis

GraphPad Prism 8 was used for analysis. The differences between two groups or more than two groups were compared using Student’s t-test. Survival analyses were conducted by Kaplan–Meier curve and log-rank test. The linear regression test was used to analyze the genes expression correlation. *P* < 0.05 was regarded as statistically significant. All the data are showed as mean ± SD.

## Results

### HnRNPK is frequently overexpressed in prostate tissues and cell lines

**Fig. 1:**
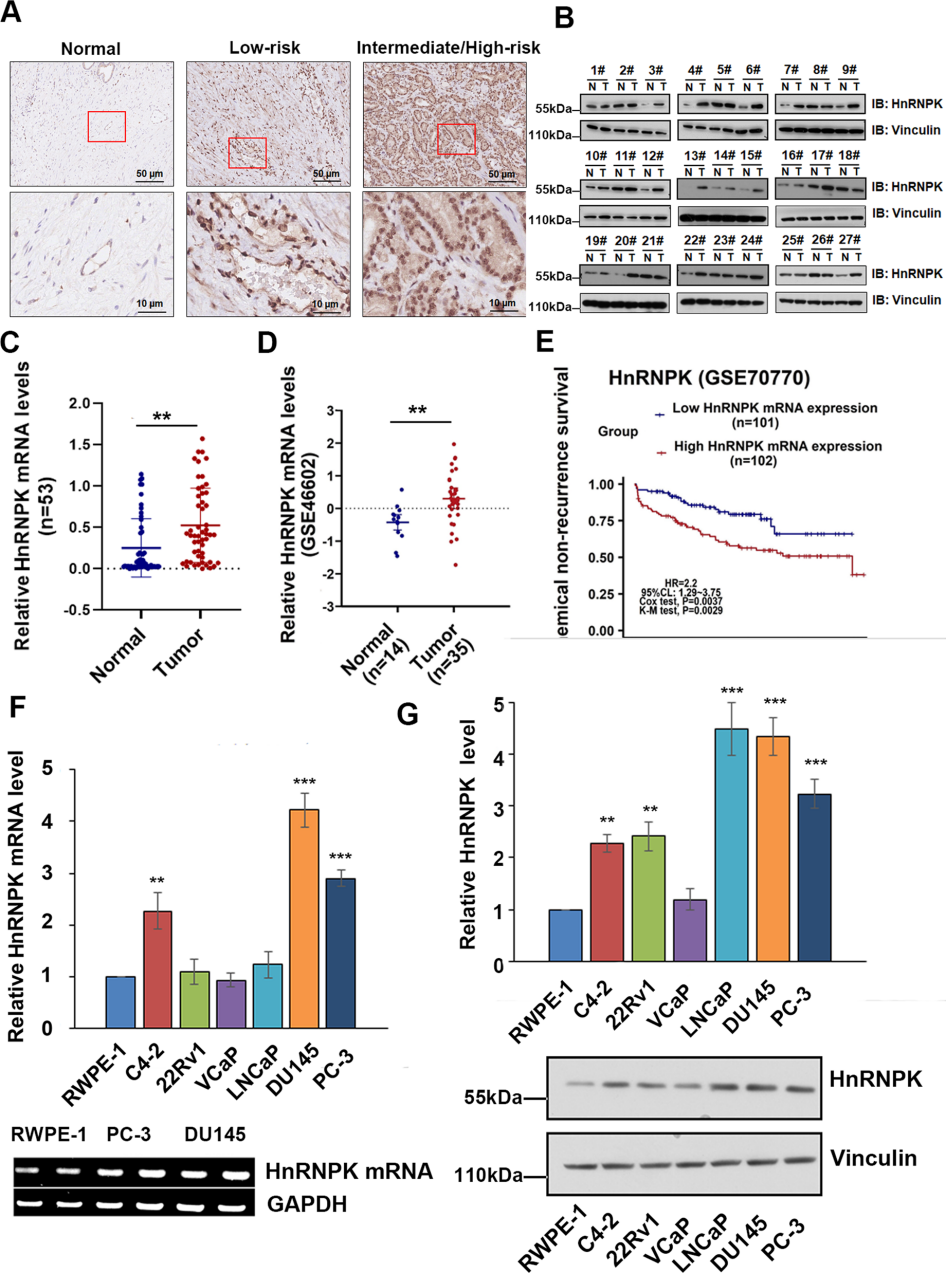
HnRNPK is frequently overexpressed in prostate cancer. **A** Twenty-two formalin-fixed and paraffin-embedded primary prostate cancer tissues were subjected to IHC analyses of the HnRNPK protein. Representative images are shown in normal, low-risk, and intermediate/high-risk prostate cancer tissues. Magnification: × 400 (top) and × 1000 (bottom). **B** Immunoblot analysis of the HnRNPK protein levels in 27 randomly selected PrCa tissues and paired noncancerous prostate tissues; vinculin was used as an internal control. **C** HnRNPK mRNA levels in 53 PrCa tissues and paired noncancerous prostate tissues. GAPDH served as loading controls. **D** Relative HnRNPK mRNA expression levels in PrCa tissues and adjacent normal prostate tissues in a public dataset (GSE70770). **E** The effect of the HnRNPK expression level on biochemical recurrence in 203 prostate cancer patients who did not undergo androgen deprivation therapy, chemotherapy, radiotherapy or other anticancer treatments was analyzed, and Kaplan–Meier plots were generated using Kaplan–Meier Plotter. **F** The level of HnRNPK in human prostate cancer cells was detected by RT–qPCR and immunoblot (**G**). *P < 0.05, **P < 0.01, and ***P < 0.001

To detect HnRNPK protein levels in PrCa, we first performed IHC analysis on 22 primary prostate adenocarcinomas. We found that HnRNPK was mainly located in the nucleus of PrCa cells, and its levels were high in 13 cases (59.1%). Moreover, HnRNPK expression was higher in intermediate/high-risk tissues than in low-risk tissues (Fig. 1A). Furthermore, we analyzed HnRNPK expression in lysates from 27 freshly harvested tissue samples of PrCa patients by immunoblotting compared with matched noncancerous tissues. Among 27 randomly selected PrCa and paired noncancerous prostate tissues, 15 tumors (55.6%) showed an increase in HnRNPK protein (Fig. 1B). Moreover, we detected HnRNPK mRNA expression in 53 paired PrCa tissues, and its levels were significantly higher than those in adjacent noncancerous prostate tissues (Fig. 1C). Additionally, a public dataset (Gene Expression Omnibus, GSE70770) containing 35 PrCa tissues and 14 normal prostate tissues also showed that HnRNPK mRNA expression was upregulated in PrCa tissues (Fig. 1D). To further investigate the clinicopathological and prognostic significance of HnRNPK levels in PrCa patients, the mRNA levels of HnRNPK in the above cohort of 53 PrCa tissues with the absence of androgen deprivation therapy, chemotherapy, radiotherapy or other anticancer treatments before surgery were classified according to age, serum PSA, Gleason score, pT stage, lymph node metastasis, seminal vesicle invasion and biochemical recurrence. We found that high HnRNPK mRNA expression was significantly associated with a higher Gleason score and biochemical recurrence (*P* < 0.05, Additional file 1: Table S1). This prognostic value was also confirmed using a larger cohort of 203 PrCa patients retrieved from the GSE70770 database, and high expression of HnRNPK was associated with higher biochemical recurrence rates (*P* < 0.05, Fig. 1E). As shown in Fig. 1F and G, HnRNPK mRNA and protein expression was remarkably high in the C4-2, DU145 and PC-3 cell lines compared with the normal prostate epithelial cell line RWPE-1 or other PrCa cell lines. Thus, HnRNPK expression was frequently higher in PrCa tissues and cell lines than in normal tissues and cell lines, predicting poor prognosis in PrCa patients.

### Knockdown of HnRNPK inhibits cell growth and cell cycle progression of prostate cells

**Fig. 2:**
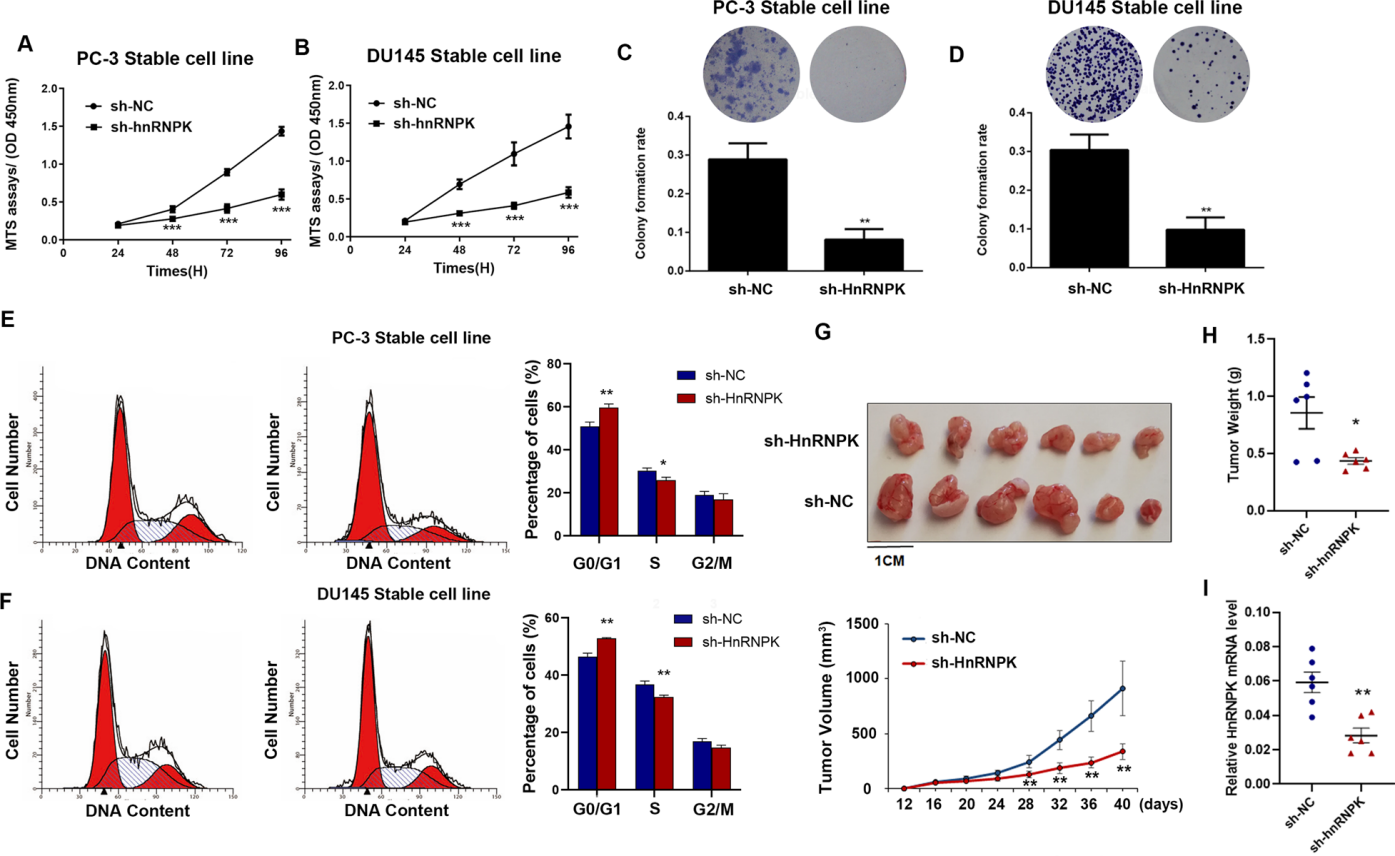
Knockdown of HnRNPK inhibits cell growth and the cell cycle in vitro and in vivo. HnRNPK was knocked down using shRNAs in PC-3 and DU145 cells. **A**, **B** MTS assays revealed cell viability curves of both stable PrCa cell lines every 24 h. **C**, **D** Representative micrographs and relative quantification of crystal violet-stained cell colonies analyzed by colony formation assay. **E**, **F** Flow cytometric analysis of PrCa cell lines (HnRNPK-silenced cells vs. NC cells). Cells were harvested at 72 h after transfection and stained with propidium iodide. The percentage of cells in each cell cycle phase is shown in the inset of each panel. **G** HnRNPK-silenced DU145 cell xenografts in nude mice (n = 6) at the experimental endpoint; tumors were dissected and photographed as shown. Tumor growth curves in mice inoculated with the indicated cells on the indicated days. **H** Each tumor formed was weighed. **I** HnRNPK mRNA expression in tumors was detected by qRT–PCR analysis. The results are plotted as the mean ± SD of three independent experiments. *P < 0.05, **P < 0.01, and ***P < 0.001

To determine the biological functions of HnRNPK, we first performed loss-of-function experiments and knocked down HnRNPK by using short hairpin RNAs (shRNAs) in PC-3 and DU145 cells. The efficiency of knockdown was confirmed by mRNA and protein levels with RT–qPCR and immunoblots (Additional file 3: Fig. S1). The results of the MTS assay showed that the proliferation capacity of PC-3 and DU145 cells was significantly reduced after silencing HnRNPK expression (Fig. 2A and B). Interestingly, the proliferation capacity was also inhibited in RWPE-1 cells by knockdown of HnRNPK expression (Additional file 4: Fig. S2). Colony formation assays further confirmed significant inhibition of cellular growth in both PrCa cell lines following HnRNPK silencing (Fig. 2C and D). Moreover, the flow cytometry results indicated that the proportion of cells in the G0/G1 phase was significantly higher and the proportion of cells in the S phase was significantly lower in HnRNPK-silenced cells than in the control cells (Fig. 2E and F). Consistently, control sh-NC DU145 cells and the corresponding stable HnRNPK-silenced cells were inoculated into BALB/C athymic mice. As shown in Fig. 2G and H, tumors formed by the HnRNPK-silenced cells were retarded in size and weight compared with those formed from the control cells. Then, the relative expression of HnRNPK mRNA in xenograft tumor tissue was verified by RT–qPCR (Fig. 2I). Collectively, these results indicate that HnRNPK inhibits PrCa cell proliferation in vitro and in vivo via its effects on the cell cycle and may play oncogenic roles in prostate cancer progression.

### miR-206 and miR-613 expression are downregulated in prostate tissues and cell lines and directly targets HnRNPK

**Fig. 3:**
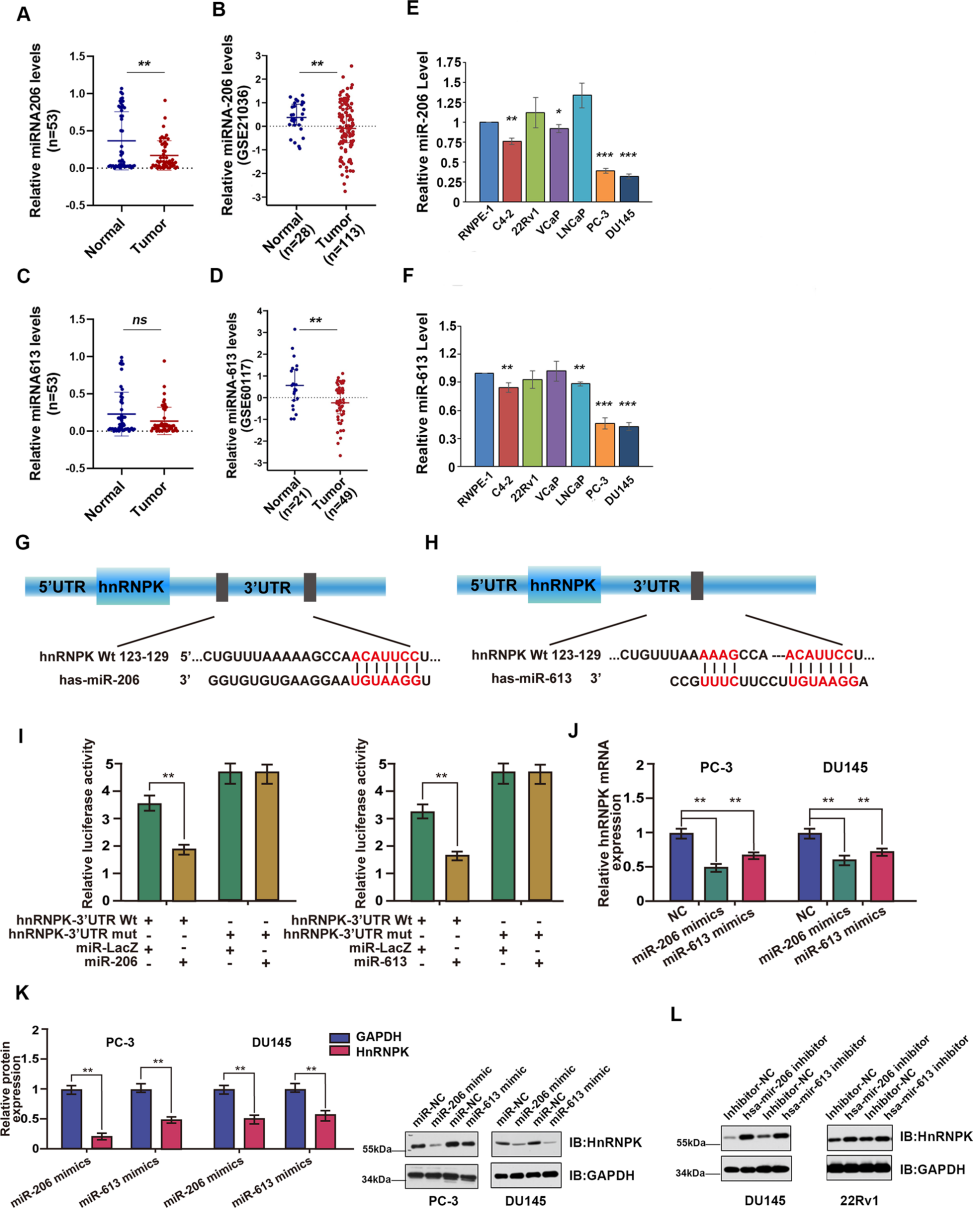
Mature miR-206 and miR-613 expression are decreased in PrCa and directly target the 3’-UTR of HnRNPK to decrease its expression. **A** Relative miR-206 and miR-613 (**C**) levels in 53 PrCa tissues and paired noncancerous prostate tissues. U6 served as a loading control. **B** Scatter diagram showing relative miR-206 and miR-613 (**D**) expression in PrCa tissues and adjacent normal prostate tissues from a public dataset (GSE21036 and GSE60117). **E** RT–qPCR analysis of relative miR-206 and miR-613 (**F**) expression in human PrCa cell lines. **G**, **H** Schematic diagram of the predicted target binding sites of miR-206 and miR-613 in the 3’-UTR of HnRNPK. The seed recognition site is denoted. The nucleotides of the 3’-UTR of HnRNPK that binds with miR-206 and miR-613 are highly conserved across species, as predicted by TargetScan (http://www.targetscan.org/vert_71/). **I** The luciferase activity of the wild-type HnRNPK 3’-UTR (Wt) and mutant HnRNPK 3’-UTR (Mut) cotransfected with miR-206/miR-613 mimics or a miRNA negative control (miR-LacZ) was measured in DU145 cells. Relative luciferase activity was plotted as the mean ± SD of three independent experiments. **J**, **K** Expression of HnRNPK in PrCa cell lines transfected with miR-206/miR-613 mimics or inhibitor (**L**) was detected by RT–qPCR and immunoblot analysis, respectively. Error bars represent the mean ± S. D of three independent experiments. *P < 0.05, **P < 0.01, ***P < 0.001

In general, miRNAs function by regulating expression of their downstream target gene(s). In this study, miR-206 and miR-613 expression were found to be downregulated significantly among the 53 randomly selected paired tissues from primary PrCa patients (Same cases above-mentioned)when compared with paired noncancerous tissues (Fig. 3A and C), which was further confirmed using two public datasets,, GSE21036 and GSE60117, respectively (Fig. 3B and D). Moreover, we analyzed miR-206 and miR-613 mRNA expression in six PrCa cell lines and found that miR-206 in the C4-2, VCap, PC-3 and DU145 cell lines (Fig. 3E) and miR-613 in the C4-2, LNCap, PC-3 and DU145 cell lines (Fig. 3F) had lower levels than in the normal prostatic cell line RWPE-1. Further putative miR-206 and miR-613 targets were predicted using TargetScan7.1 algorithms, a bioinformatic tool and found both of them could bind to the 3’-UTR of HnRNPK mRNA(Fig. 3G and H). Therefore, it is possible that both miRNAs inhibit HnRNPK expression in PrCa tissues and cell lines by directly binding to its mRNA 3’-UTR. By employing a dual-luciferase reporter system, we subcloned the 3’-UTR of HnRNPK mRNA, including the predicted miR-206 and miR-613 recognition site (Wt), or the mutated sequences (Mut) into the pGL3 vector downstream of the luciferase open reading frame. miR-LacZ as a miRNA blank vector control. As shown in Fig. 3I, miR-206 and miR-613 inhibited the activity of luciferase with the wild-type but not mutant 3’-UTR of HnRNPK mRNA in DU145 cells. We further identified whether miR-206 or miR-613 negatively regulated the expression of HnRNPK in PrCa. Our data first confirmed the efficiency of overexpression and knockdown of miR-206 and miR-613 in PrCa cells transfected with their corresponding mimics or inhibitor, respectively (Additional file 5: Fig. S3). Then, in line with the expression of miR-206 and miR-613, the levels of HnRNPK mRNA and protein showed a tendency toward an inverse correlation, as determined using RT–qPCR and immunoblot analysis (Fig. 3J–L). Taken together, these data support the bioinformatic prediction suggesting that the 3’-UTR of HnRNPK is a direct target of miR-206 and miR-613.

### *Overexpression of miR-206/miR-613 can inhibit PrCa cell proliferation *in vitro* and vivo*

**Fig. 4:**
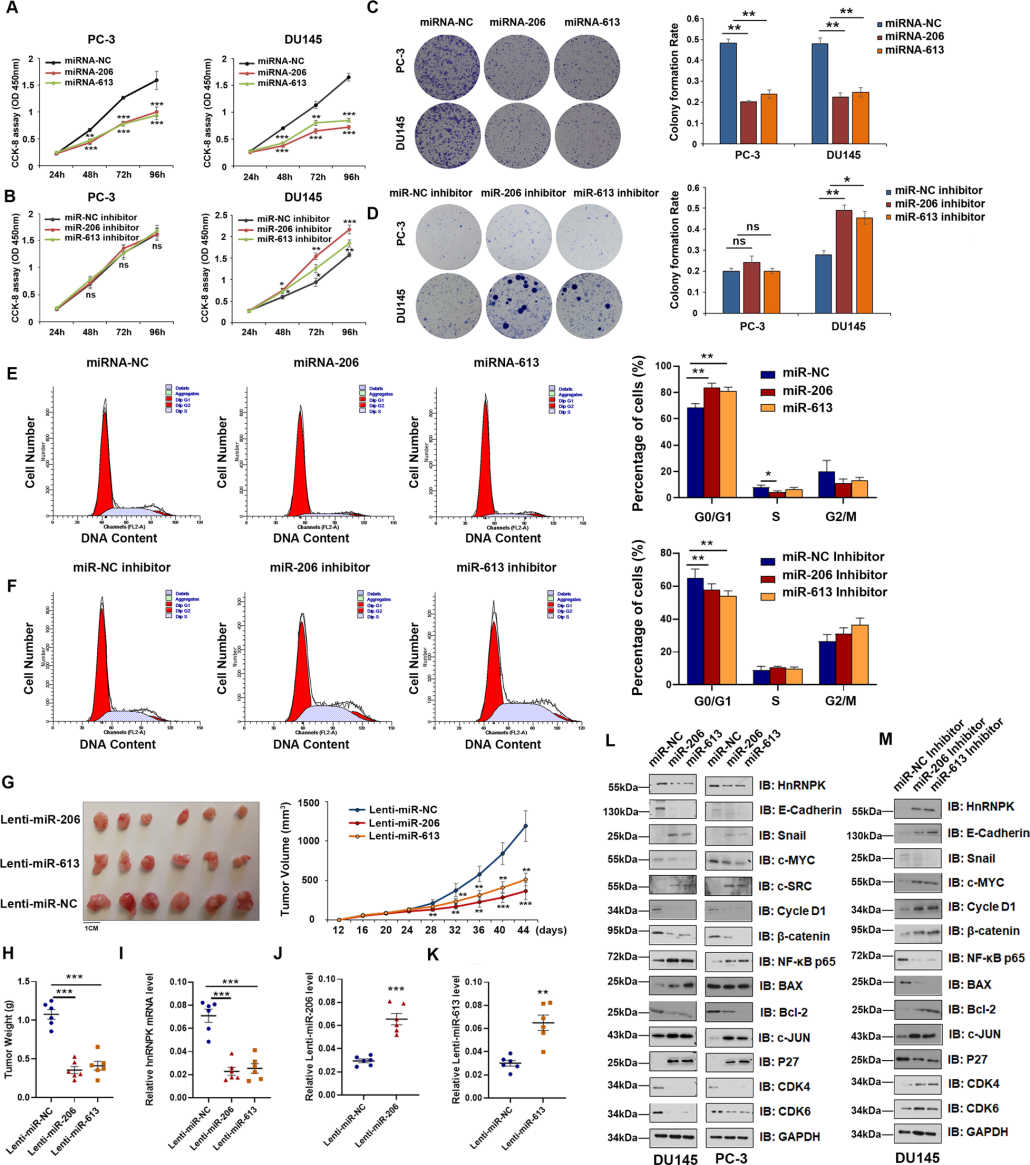
Overexpression of miR-206 and miR-613 inhibits cell growth and the cell cycle in vitro and in vivo. **A**, **B** CCK-8 assay of cell viability in PrCa cell lines transfected with miR-206 and miR-613 mimics or inhibitors at 24, 48, 72 and 96 h. **C** Representative micrographs and **D** relative quantification of crystal violet-stained cell colonies analyzed by a colony formation assay. **E**, **F** Flow cytometric analysis of DU145 cell lines. Cells were harvested at 72 h after transfection with the indicated miRNA and stained with propidium iodide. The percentage of cells in each cell cycle phase is shown in the inset of each panel. **G** miR-206 and miR-613-overexpressing DU145 cell xenografts in nude mice (n = 6) at the experimental endpoint; tumors were dissected and photographed as shown. Tumor growth curves in mice inoculated with the indicated cells on the indicated days. **H** Each tumor formed was weighed. **I**–**K** HnRNPK mRNA and miR-206 and miR-613 expression in tumors was detected by qRT–PCR analysis. **L**, **M** PC-3 and DU145 cells were treated with or without miR-206, miR-613 mimics or the miR-206, miR-613 inhibitor for 72 h, respectively. The expression levels of the indicated proteins were analyzed by immunoblotting. The results were plotted as the mean ± SD of three independent experiments. *P < 0.05, **P < 0.01, and ***P < 0.001

Next, we sought to understand the biological effects of miR-206 and miR-613 in PrCa. We induced overexpression of miR-206/miR-613 using miR-206/miR-613 mimics and silenced miR-206/miR-613 using a miR-206/miR-613 inhibitor in PrCa cells and studied the effects on cell growth. The CCK-8 and colony formation assays showed that PrCa cells overexpressing miR-206 or miR-613 had significantly lower proliferation ability than the control cells (Fig. 4A and C). In contrast, silencing of endogenous miR-206 or miR-613 led to a significantly higher proliferation rate than that observed in the control cells, with the exception of the PC-3 cell lines (Fig. 4B and D). Next, the flow cytometry results indicated that overexpressing miR-206 or miR-613 dramatically increased the cell population in the G0/G1 phase, whereas it reduced the cell population in the S and G2/M phases (Fig. 4E). In contrast, depletion of endogenous miR-206 or miR-613 decreased the cell population in the G0/G1 phase and increased the cell population in the S and G2/M phases in DU145 cells (Fig. 4F). Consistently, induction of miR-206 or miR-613 by shRNA in DU145 cells significantly retarded tumor growth in xenograft mouse models (Fig. 4G and H). Then, the relative expression of HnRNPK mRNA, miR-206 and miR-613 in tumor tissue was verified by RT–qPCR (Fig. 4I–K). Taken together, miR-206 or miR-613 could exert a significant inhibitory effect on tumorigenesis by repressing HnRNPK in vitro and in vivo.

In line with the results for HnRNPK-silenced cells mediated by miR-206 or miR-613, the protein abundance of Cycle D1, CDK4, and CDK6, which promote the cell cycle, was significantly decreased, whereas the protein abundance of P27, which arrests the cell cycle, was increased compared with that in the control cells. Moreover, depletion of endogenous HnRNPK significantly upregulated the protein levels of apoptosis, such as that of BAX and c-JUN, and inhibited the antiapoptotic protein levels of BCL-2 and the oncogene c-MYC. Interestingly, HnRNPK-silenced cells also exhibited increased protein abundance of the transcription factor Snail and oncogene NF-Κb/p65, c-SRC and decreased biomarkers of EMT, such as E-cadherin and β catenin (Fig. 4L). In contrast, HnRNPK-upregulated cells mediated by miR-206 or miR-613 inhibitor showed an inverse tendency for the aforementioned protein abundance (Fig. 4M). The correlation network dataset (GSE 88808) also indicated that HnRNPK shared a similar correlation with the aforementioned proteins (Additional file 6: Fig. S4). These data strongly suggest that HnRNPK regulates several biological functions crucial for PrCa development, including proliferation, apoptosis and EMT.

### ***Cul3***^***SPOP***^*** E3 ligase degrades HnRNPK protein***

**Fig. 5:**
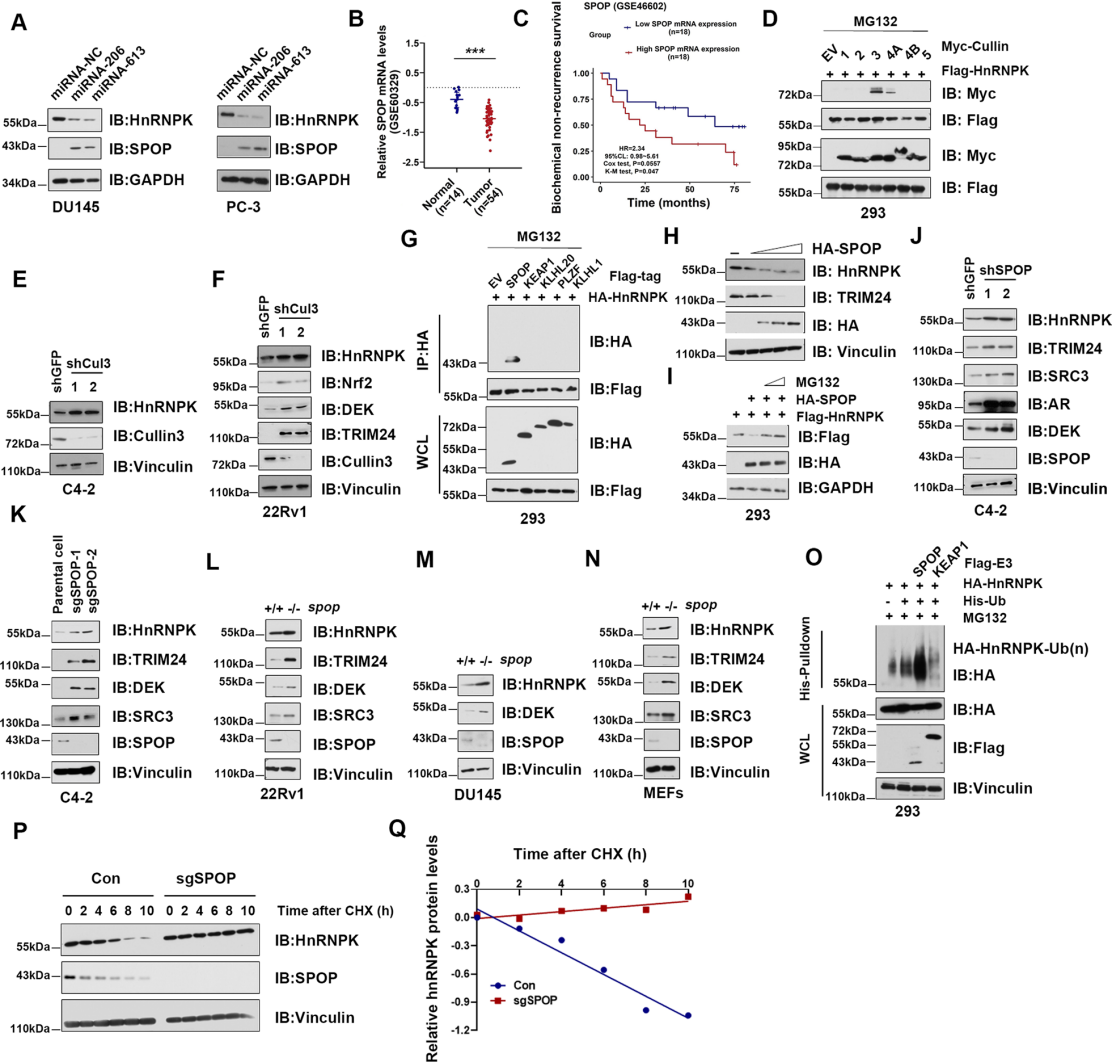
The Cullin 3 SPOP E3 ubiquitin ligase negatively regulates the stability of HnRNPK. **A** Expression of HnRNPK and SPOP in PrCa cells transfected with miR-206 and miR-613 mimics was detected by Immunoblot analysis. **B** Relative SPOP mRNA expression levels in PrCa tissues and adjacent normal prostate tissues in a public data set (GSE60329). **C** Effect of the SPOP expression level on Biochemical recurrence in 36 prostate cancer patients who did not undergo androgen deprivation therapy, chemotherapy, radiotherapy or other anticancer treatment was analyzed, and Kaplan–Meier plots were generated using a Kaplan–Meier Plotter. **D** WB analysis of WCL and immunoprecipitates (IP) derived from 293 cells transfected with Flag-HnRNPK and various Myc-tagged Cullin constructs. 30 h post-transfection, cells were treated with 10 μM MG132 for 10 h before harvesting. **E** WB analysis of WCL derived from C4-2 and 22Rv1 (**F**) cells infected with the indicated lentiviral shRNAs. Infected cells were selected with 1 μg/ml puromycin for 72 h to eliminate non-infected cells before harvesting. **G** WB analysis of WCL and IP derived from 293 cells transfected with HA-HnRNPK and Flag-tagged BTB domain-containing protein constructs. 30 h post-transfection, cells were treated with 10 μM MG132 for 10 h before harvesting. EV, empty vector. **H** WB analysis of WCL derived from C4-2 or 293 **I** cells transfected with increasing doses (0.5–3 μg) of indicated plasmids. Where indicated, 10 µM MG132 was added for 10 h before harvesting. **J** WB analysis of WCL derived from C4-2 cells infected with the indicated lentiviral shRNAs. Infected cells were selected with 1 μg/ml puromycin for 72 h to eliminate non-infected cells before harvesting. **K**–**N** IB analysis of WCL derived from C4-2, 22Rv1, DU145 and mouse embryonic fibroblasts (MEFs) cells with SPOP knockout by the CRISPR technology. **O** WB analysis of WCLs and His pull-down products derived from 293 cells transfected with indicated constructs and treated with MG132 (10 μM) 10 h. **P** SPOP knockout cells (sg SPOP) as well as parental C4-2 cells (Con) were treated with 100 μg/ml cycloheximide (CHX), and cells were harvested at the indicated time points. Relative HnRNPK protein abundance was quantified by Image J and plotted in **Q**. Data was shown as mean ± SD for three independent experiments. *P < 0.05, **P < 0.01, ***P < 0.001

The findings above have revealed a new posttranscriptional mechanism of HnRNPK regulation via miR-206 and miR-613 that thereby represses PrCa cell proliferation. Interestingly, the HnRNPK-silenced PrCa cells mediated by miR-206 and miR-613 led to higher SPOP expression than that in the control cells (Fig. 5A). In addition, SPOP mRNA expression in the correlation network dataset (GSE 88808) also showed a tendency toward a strongly inverse correlation with HnRNPK in PrCa (Additional file 6: Fig. S4). Two public datasets further confirmed that SPOP mRNA expression was significantly downregulated in human PrCa tissues (GSE60329) (*P* < 0.05, Fig. 5B) and the SPOP expression showed a tendency toward an inverse correlation with the recurrence rates (GSE46602) (*P* < 0.05, Fig. 5C).

SPOP is an adaptor protein of Cullin 3-based E3 ubiquitin complexes, has been shown to participate in diverse cellular processes and plays tumor suppressive roles in PrCa [20, 22], suggesting that HnRNPK may be a substrate of SPOP. To uncover the underlying regulatory mechanisms, we first screened a panel of Cullin scaffolding proteins to identify the potential E3 complex for HnRNPK. Cullin 3, and to a much lesser extent, Cullin 4A, but not other Cullin family members, specifically interacted with HnRNPK in cells (Fig. 5D). Consistently, deletion of endogenous Cullin 3 in PrCa cell lines, including C4-2 and 22Rv1, dramatically upregulated the protein abundance of endogenous HnRNPK (Fig. 5E and F). Previous studies demonstrated that Cullin 3 selectively recruits downstream substrates via interaction with BTB domain-containing proteins as substrate-specific adaptors, including but not limited to SPOP, KEAP1, KLHL1, PLZF and KLHL12 [25]. However, we found that only SPOP, and not the other Cullin 3-based adaptor proteins we examined, specifically interacts with HnRNPK (Fig. 5G). Notably, SPOP promoted HnRNPK degradation in a dose-dependent manner in PrCa cells (Fig. 5H). This process could be efficiently blocked by 10 μM MG132 for 10 h before harvesting (Fig. 5I), indicating that SPOP can regulate HnRNPK abundance through the posttranslational ubiquitin–proteasome pathway. Consistent with these findings, depletion of endogenous SPOP in multiple human PrCa cell lines or MEFs resulted in an increase in HnRNPK abundance as well as other identified SPOP substrates, including TRIM24, SRC3, AR and DEK (Fig. 5J–N). Importantly, we found that SPOP specifically promotes HnRNPK ubiquitination in cells (Fig. 5O). Then, the half-life of HnRNPK was significantly extended in SPOP-knockout cells (Fig. 5P and Q). Taken together, our results suggest that the Cullin 3/SPOP E3 ligase complex specifically regulates HnRNPK stability.

### Patients-associated SPOP mutants are incapable of degrading HnRNPK

**Fig. 6:**
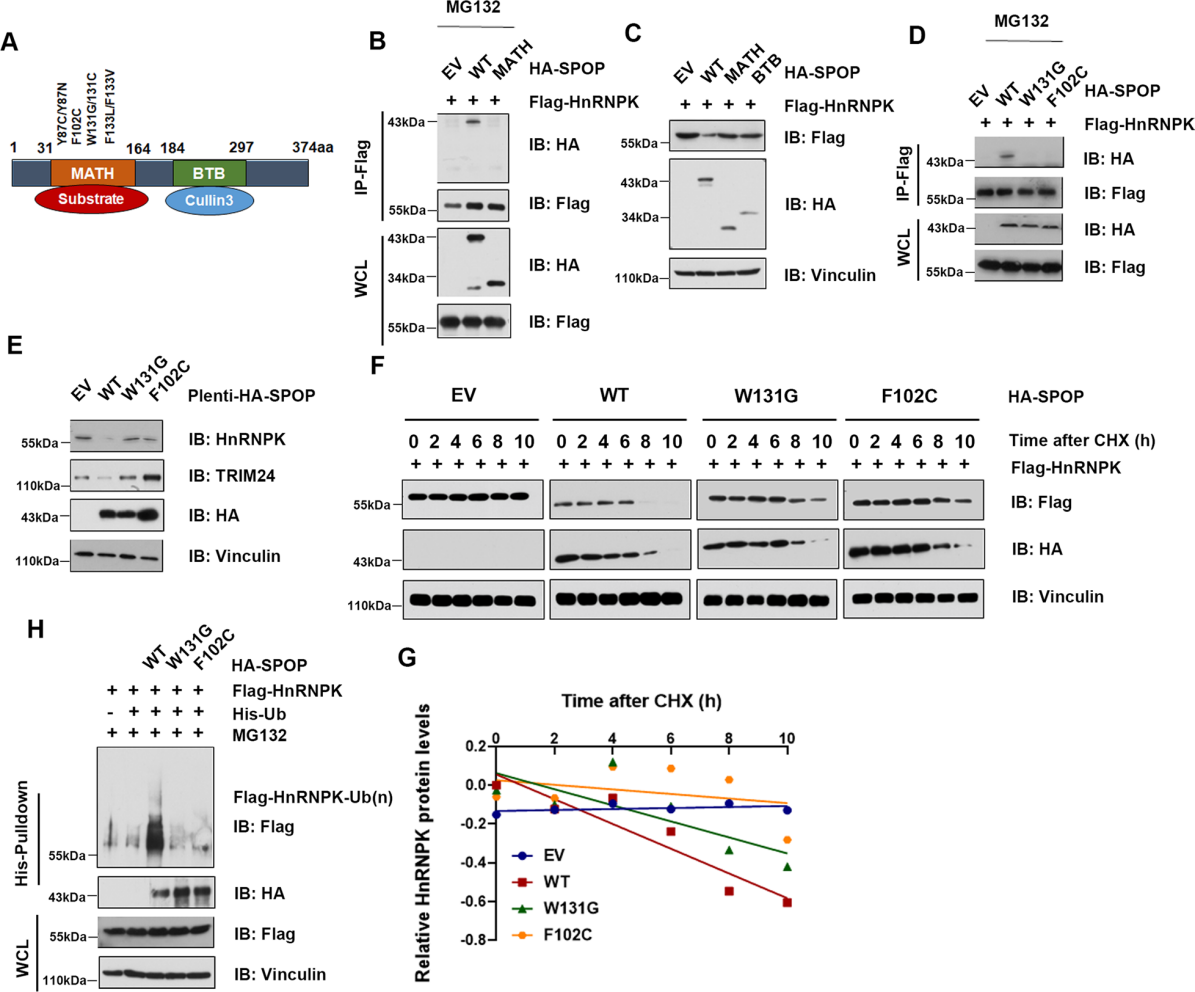
Prostate cancer-associated SPOP mutants impair to interact with and degrade HnRNPK. **A** A schematic illustration of SPOP domains and prostate cancer-associated mutations. **B** WB analysis of WCL and immunoprecipitates derived from 293 cells transfected with Flag-HnRNPK and HA-SPOP-WT or deletion of MATH domain-SPOP constructs. 30 h post-transfection, cells were treated with 10 μM MG132 for 10 h before harvesting. EV, empty vector. **C** WB analysis of WCL derived from 293 cells transfected with indicated plasmids. **D** WB analysis of WCL and IP derived from 293 cells transfected with HA-SPOP-WT or prostate cancer -associated SPOP mutants. Cells were treated with 10 μM MG132 for 10 h before harvesting. **E** WB analysis of WCL derived from C4-2 cells stably expressing HA-SPOP-WT or PrCa-associated SPOP mutants. **F** 293 cells transfected with Flag-HnRNPK together with the indicated HA-SPOP expressing plasmids. 30 h post-transfection, cells were treated with 100 μg/ml CHX for the indicated time period before harvesting. Relative Flag-HnRNPK protein abundance was quantified by Image J and plotted in (**G**). **H **WB analysis of WCLs and His pull-down products derived from 293 cells transfected with indicated constructs and treated with MG132 (10 μM) 10 h

SPOP is a member of the MATH-BTB protein family containing an N-terminal MATH domain and a C-terminal BTB domain [25]. Most of the somatic SPOP mutations detected thus far in PrCa such as Y87C, F102C, W131G and F133V, exclusively occur in the MATH domain, which is responsible for substrate recognition and interaction [21] (Fig. 6A). We postulated that patients-associated SPOP mutants may be defective in mediating HnRNPK ubiquitination. To address this, we first found that deletion of the MATH domain abolishes the SPOP interaction with HnRNPK by co-IP assays (Fig. 6B), and then the MATH domain and BTB domain are both required for SPOP-mediated HnRNPK degradation (Fig. 6C). In keeping with the above finding, we next determined whether SPOP mutants observed in PrCa impair HnRNPK stability. We ectopically expressed two MATH domain-mutated SPOPs (W131G and F102C) that are frequently observed in PrCa. As shown in Fig. 6D, mutations of the residues at the MATH domain abrogated the ability of SPOP to interact with HnRNPK and thereby failed to promote the degradation of HnRNPK compared with wild-type SPOP (Fig. 6E). Consistently, ectopic expression of SPOP mutants, compared with wild-type SPOP, extended the half-life of HnRNPK (Fig. 6F and G) and was largely deficient in promoting HnRNPK polyubiquitination according to the in vivo ubiquitination assay (Fig. 6H). These data indicate that patient-associated SPOP mutants lost the capacity to promote ubiquitination and destruction of HnRNPK, therefore partially providing a molecular mechanism to explain the aberrant accumulation of HnRNPK in prostate cancer cells and tissue.

## Discussion

**Fig. 7:**
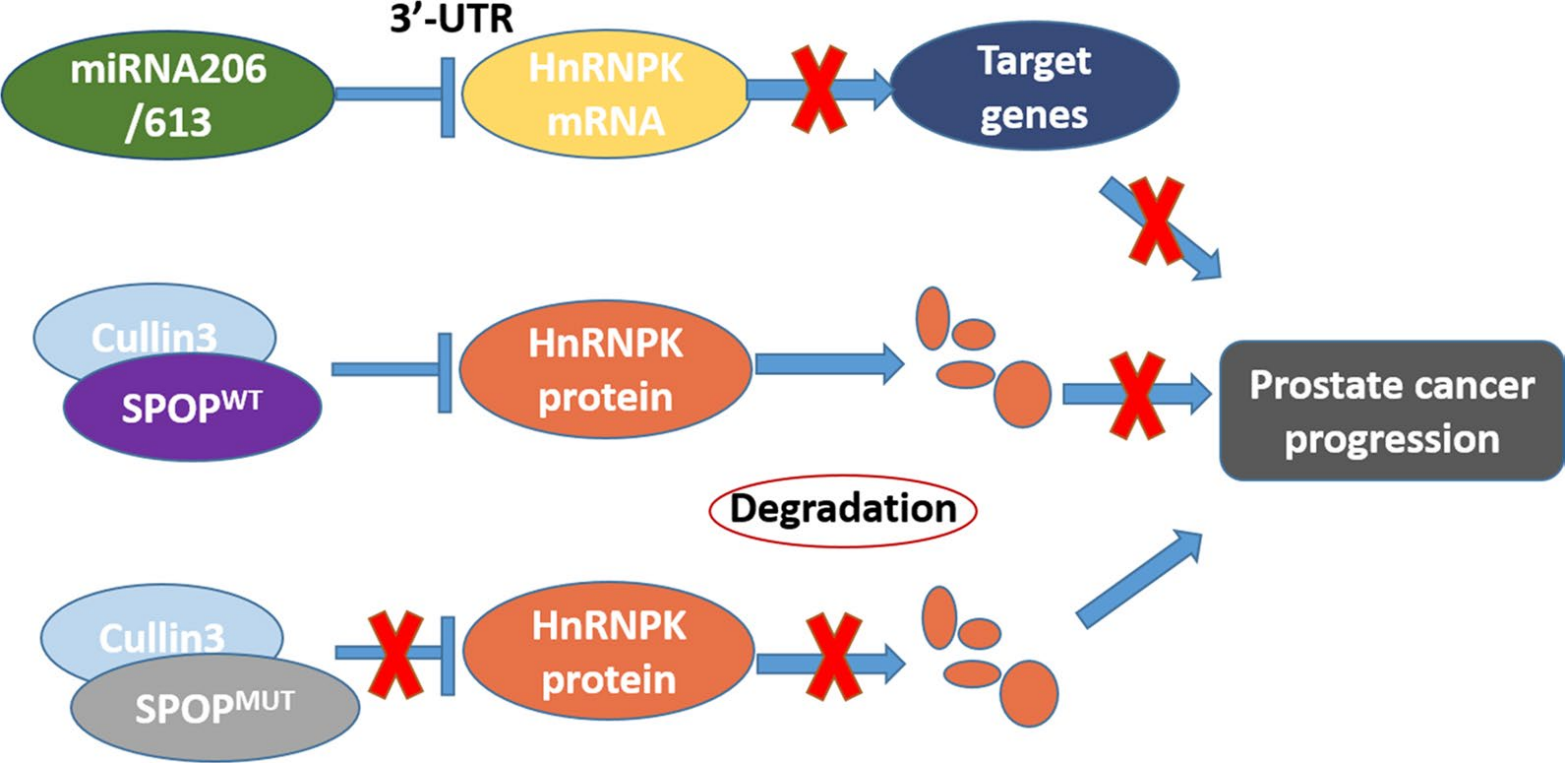
A schematic diagram deciphering the mechanism for the transcriptional regulation and ubiquitination dependent regulation of HnRNPK Oncogenic Function in prostate tumorigenesis

Our novel discovery highlights the oncogenic role of HnRNPK in PrCa, and miR-206 or miR-613 inhibits HnRNPK expression at the posttranscriptional level by directly targeting the HnRNPK mRNA 3’-UTR and thereby repressing PrCa cell carcinogenesis. This miRNA-mediated downregulation of HnRNPK provides new insight into therapeutic strategies for PrCa. On the other hand, our studies delineated the upstream posttranslational regulatory mechanisms of HnRNPK by SPOP-mediated polyubiquitination and subsequent degradation in PrCa (Fig. 7). Hence, we envision that our studies will provide the rationale for developing an optimal treatment strategy based on individual tumor genetic status for individual PrCa patients.

Posttranscriptional gene regulation (PTGR) is involved in the precise control of many oncogenes and tumor-suppressing genes. As trans-acting factors and tumor suppressors or oncogenes, miRNAs play key roles in PTGR mainly through interactions with the 3’-UTR of target mRNAs in various human cancers (cis-elements) [26]. In this study, we focused on PrCa cells and tissues to determine whether miRNAs can epigenetically influence HnRNPK expression. Intriguingly, the expression of miR-206 and miR-613 was downregulated and inversely correlated with HnRNPK expression in PrCa tissues and in the majority of PrCa cell lines, implying that miR-206 and miR-613 might functionally contribute to the expression of HnRNPK in PrCa. Therefore, we performed in silico prediction of microRNA targets and found that miR-206 and miR-613 can both potentially bind to target sites of the HnRNPK 3’-UTR. Then, we used a luciferase reporter assay to confirm that HnRNPK is a bona fide target of miR-206 and that miR-613 negatively regulates the expression of HnRNPK at the posttranscriptional level. In functional studies, we assessed the effect of miR-206/miR-613 and HnRNPK on PrCa growth both in vitro and in vivo. miR-206/miR-613 overexpression significantly repressed the proliferation and colony formation of tumor cells in vitro. These effects appeared to be mediated by inhibition of the HnRNPK oncogene, leading to tumor cell arrest at the G1 phase of the cell cycle. Whereas silencing of endogenous miR-206 or miR-613 in PC-3 cell lines did not show the higher proliferation rate (Fig. 4B and D), indicating the possibility that miR-206 or miR-613 may have different functions in different cellular contexts.

Furthermore, transfection of miR-206/miR-613 effectively suppressed the tumorigenicity of PrCa cells in a nude mouse model. Supporting our findings, recent studies have found that HnRNPK downregulation suppresses cell proliferation in pancreatic cancer [27]. However, the underlying mechanism remains largely unknown. We further determined that HnRNPK regulates the cell cycle of PrCa cells mainly by transcriptional regulation of Cycle D1, CDK4, CDK6 and P27. In addition, we first demonstrated that HnRNPK maintained antiapoptotic effects in PrCa cells via transcriptional regulation of BAX, c-JUN and BCL-2. Similarly, HnRNPK suppresses apoptosis independent of p53 status in hepatocellular carcinoma by increasing XIAP transcription [28], suggesting that the mechanism of HnRNPK in apoptosis differs between cancers. Moreover, new studies have found that HnRNPK transcription and translation activate several important oncogenes, including c-MYC [6, 29], and inhibit its interaction with c-Src [30]. Consistent with our findings, c-MYC was activated, whereas c-SRC was inhibited via downregulated miR-206/613 in prostate cancer cell. These results suggest that HnRNPK plays an oncogenic role in PrCa by directly mediating these genes. Yet it's worth noting that the level of NF-KB p65 and Snail increased after miRNAs-mediated HnRNPK silencing, along with decreased E-cadherin and β catenin, which theoretically can promote EMT and inhibit apoptosis for PrCa cell. Several studies have showed NF-κB complexes are capable of promoting PrCa progression to CRPC and associated with the metastatic phenotype, which process is accompanied by EMT pathological feature changes [31, 32]. Thus, whether HnRNPK can also play a potential role in oncogenic pathway in CRPC pathogenesis is needed to further elucidate.

HnRNPK is subject to several posttranslational modifications, such as methylation [33] and sumoylation [34], which can regulate its interactions with different molecules and influence its functions. However, the ubiquitination and degradation of HnRNPK in normal condition or other tissue contexts, especially in PrCa are not well investigated. We first provide experimental evidence demonstrating that the E3 ubiquitin ligase SPOP plays a critical tumor suppressive role in PrCa by specifically binding and promoting the degradation of HnRNPK via polyubiquitination. Interestingly, previous studies have reported that HnRNPK is stabilized following DNA damage through the inhibition of its HDM2-mediated ubiquitin-dependent degradation in SAOS2 and U2OS cells [35]. However, induction of HnRNPK in response to DNA damage and ubiquitylation of HnRNPK mediated by wild-type HDM2 was not generally seen in any panel of cancer cell lines, suggesting that each E3 ligase could plausibly work in different cell and cell cycle phases or in a temporal or spatial specificity to provide timely control of HnRNPK stability.

Although prostate cancer has been associated with a low mutational burden, one of the most common recurrently mutated genes is Cullin3^SPOP^ [18, 36]. SPOP mutations may represent a distinct subtype of PrCa, as it lacks other genetic changes, such as *PTEN* and *PIK3CA* alterations or TP53 mutations. In addition, the SPOP protein plays a role as a tumor suppressor that negatively regulates the stability of multiple other oncogenic substrates in PrCa, including AR, SRC-3, c-MYC, ERG, DEK, BRD4 and Trim24 [19–22, 37–39]. Mutations have been identified as early and divergent driver events in prostate carcinogenesis. Notably, our findings show that HnRNPK knockdown can impair the cell growth and cell cycle progression of PrCa cell lines, and prostate cancer-associated SPOP mutants, including F102C and W131G, which are clustered in its substrate-recruiting MATH domain, restrain its capability to bind and promote HnRNPK polyubiquitination and degradation. Thus, our current study provides a possible mechanism to explain why HnRNPK is overexpressed in PrCa, in part by evading SPOP-mediated degradation, and suggests that HnRNPK inhibition may be an intervention strategy for SPOP-mutated PrCa. However, many more studies are warranted in the future, such as to identify the specific degron of HnRNPK as the major motif that is responsible for SPOP-dependent regulation of its stability. Moreover, consistent with a critical role for HnRNPK as a transcriptional coactivator for AR, it is worthwhile to investigate whether AR target genes and AR inhibitor sensitivity correlate with HnRNPK overexpression in somatic SPOP mutant cells.

## Conclusions

In summary, our novel discovery highlights the oncogenic role of HnRNPK in PrCa, and miR-206 or miR-613 inhibits HnRNPK expression at the posttranscriptional level by directly targeting the HnRNPK 3’-UTR and thereby repressing PrCa cell carcinogenesis. This miRNA-mediated downregulation of HnRNPK provides new insight into therapeutic strategies for PrCa. On the other hand, our studies delineated the upstream posttranslational regulatory mechanisms of HnRNPK by SPOP-mediated polyubiquitination and subsequent degradation in PrCa. Hence, we envision that our studies will provide the rationale for developing an optimal treatment strategy based on individual tumor genetic status for individual PrCa patients.

## Supplementary Information

**Additional file 1: Table S1.** Demographic and clinical characteristics of PrCa patients and the level of miRNA and hnRNPK mRNA expression in tumor tissue specimens

**Additional file 2: Table S2.** shRNA sequences and qRT‐PCR primer sequences.

**Additional file 3: Fig. S1. A and B** HnRNPK mRNA and protein levels in stable PC-3 and DU145 cells was detected by RT-qPCR and Immunoblot analysis respectively.

**Additional file 4: Fig. S2.** Knocked down HnRNPK respectively by using siRNAs in RWPE-1 cells. MTS assays revealed cell viability curves in every 24 h.

**Additional file 5: Fig. S3. A–D** Expression of miR-206, miR-613 and HnRNPK in PrCa cells transfected with corresponding miRNA mimics or inhibitor was detected by RT-qPCR and Immunoblot analysis respectively.* P < 0.05; ** P < 0.01; *** P < 0.001

**Additional file 6: Fig. S4.** The correlation network of HnRNPK in a public dataset (GSE 88808)

## Data Availability

All data generated during this study are included in this article.

## Abbreviations

miR-206: MicroRNA-206
miR-613: MicroRNA-613
PrCa: Prostate cancer
3’-UTR: 3’-Untranslated region
HnRNPK: Heterogeneous nuclear ribonucleoprotein K
AR: Androgen receptor
CRPC: Castration-resistant prostate cancer
SPOP: Speckle-type POZ protein
SRC-3: Steroid receptor coactivator 3
TRIM24: Tripartite motif-containing 24
shRNAs: Short hairpin RNAs
PTGR: Post-transcriptional gene regulation
CDK6: Cyclin dependent kinase 6
CDK4: Cyclin dependent kinase 4
EMT: Epithelial-mesenchymal transition
MEFs: Mouse embryonic fibroblasts
CHX: Cycloheximide
