# Supplementary material for: Transcriptional regulation and ubiquitination-dependent regulation of HnRNPK oncogenic function in prostate tumorigenesis
Source: Cancer Cell Int. 2021 Dec 2;21:641. doi: 10.1186/s12935-021-02331-x (PMC8641147; doi:10.1186/s12935-021-02331-x)
Supplement: Supplementary file 1 — Additional file 1: Table S1. Demographic and clinical characteristics of PrCa patients and the level of miRNA and hnRNPK mRNA expression in tumor tissue specimens [file 12935_2021_2331_MOESM1_ESM.doc]

**Table S1.** Demographic and clinical characteristics of PrCa patients and the level of miRNA and hnRNPK mRNA expression in tumor tissue specimens

| **Variables** | Number of cases (%) | hnRNPK mRNA | *P* value | miRNA206 | *P* value | miRNA613 | *P* value |
| --- | --- | --- | --- | --- | --- | --- | --- |
| **Age (years)** |  |  |  |  |  |  |  |
| >65Y | 35 (66%) | 0.5223 ± 0.1015 | 0.727 | 0.0418 ± 0.0224 | 0.635 | 0.0457 ± 0.0221 | 0.401 |
| ≤64Y | 18 (34%) | 0.5424 ± 0.0954 |  | 0.0473 ± 0.0281 |  | 0.0463 ± 0.0213 |  |
| **Serum PSA** |  |  |  |  |  |  |  |
| ≥ 10 ng/ml | 34 (64.2%) | 0.5322 ± 0.1036 | 0.764 | 0.0484 ± 0.0223 | 0.548 | 0.0453 ± 0.0257 | 0.447 |
| < 10 ng/ml | 19 (35.8%) | 0.5235 ± 0.0928 |  | 0.0475 ± 0.0236 |  | 0.0565 ± 0.0618 |  |
| **Gleason score** |  |  |  |  |  |  |  |
| ≥ 7 | 29 (54.7%) | 0.5508 ± 0.1035 | **0.0191** | 0.0495 ± 0.0238 | 0.532 | 0.0439 ± 0.0271 | 0.651 |
| < 7 | 24 (45.3%) | 0.4845 ± 0.0938 |  | 0.0476 ± 0.0257 |  | 0.0465 ± 0.0518 |  |
| **pT stage** |  |  |  |  |  |  |  |
| ≥ T3 | 28 (52.8%) | 0.5320 ± 0.1056 | 0.207 | 0.0515 ± 0.0224 | 0.285 | 0.05113 ± 0.0428 | 0.598 |
| < T3 | 25 (47.2%) | 0.4970 ± 0.0921 |  | 0.0493 ± 0.0268 |  | 0.05661 ± 0.0315 |  |
| **Lymph node metastasis** |  |  |  |  |  |  |  |
| Presence | 23 (43.4%) | 0.5417 ± 0.1014 | 0.422 | 0.0488 ± 0.0254 | 0.922 | 0.0498 ± 0.0239 | 0.339 |
| Absence | 30 (56.6%) | 0.5195 ± 0.0970 |  | 0.0526 ± 0.0255 |  | 0.0514 ± 0.0325 |  |
| **Biochemical recurrence** |  |  |  |  |  |  |  |
| Presence | 26 (49.1%) | 0.5682 ± 0.0762 | **0.0038** | 0.0498 ± 0.0239 | 0.644 | 0.0453 ± 0.0514 | 0.166 |
| Absence | 27 (50.9%) | 0.4915 ± 0.1050 |  | 0.0543 ± 0.0214 |  | 0.0508 ± 0.0436 |  |

Data are presented as mean ± SEM. Kruskal-Wallis test for comparison between three or more groups.
