# Supplementary material for: Transcriptional regulation and ubiquitination-dependent regulation of HnRNPK oncogenic function in prostate tumorigenesis
Source: Cancer Cell Int. 2021 Dec 2;21:641. doi: 10.1186/s12935-021-02331-x (PMC8641147; doi:10.1186/s12935-021-02331-x)
Supplement: Supplementary file 2 — Additional file 2: Table S2. shRNA sequences and qRT‐PCR primer sequences. [file 12935_2021_2331_MOESM2_ESM.docx]

**Table S2.** shRNA sequences and qRT‐PCR primer sequences

|  | sequences |
| --- | --- |
| HnRNPK shRNA | CCGGTGATCTTGGTGGACCTATTATCTCGAGATAATAGGTCCACCAAGATCATTTTTG |
| SPOP shRNA1 | CCGGCACAAGGCTATCTTAGCAGCTCTCGAGAGCTGCTAAGATAGCCTTGTGTTTTTTG |
| SPOP shRNA2 | CCGGCACAGATCAAGGTAGTGAAATCTCGAGATTTCACTACCTTGATCTGTGTTTTTTG |
| CULLIN3 shRNA1 | CCGGGACTATATCCAGGGCTTATTGCTCGAGCAATAAGCCCTGGATATAGTCTTTTTG |
| CULLIN3 shRNA2 | CCGGCGTGTGCCAAATGGTTTGAAACTCGAGTTTCAAACCATTTGGCACACGTTTTTG |
| GAPDH-F | 5’-TCCCATCACCATCTTCCA-3’ |
| GAPDH -R | 5’-CATCACGCCACAGTTTCC-3’ |
| HnRNPK-F | 5’-GGCAGTGATTGGAAAAGGAG -3’ |
| HnRNPK-R | 5’-CACTGCTGTCTGGGACTGAA-3’ |
| pri-miRNA-206-F | 5′-CCCAACAAGCTCTGCCTG-3′ |
| pri-miRNA-206-R | 5′-GGGAGCATAGTTGACCTGAAAC-3′ |
| pri-miRNA-613-F | 5′-GTGAGTGCGTTTCCAAGTGT-3′ |
| pri-miRNA-613-R | 5′-TGAGTGGCAAAGAAGGAACAT-3′ |
| U6-F | 5′-TTGGTCTGATCTGGCACATATAC-3′ |
| U6-R | 5′-AAAAATATGGAGCGCTTCACG-3′ |
