# Supplementary figures and images for: Transcriptional regulation and ubiquitination-dependent regulation of HnRNPK oncogenic function in prostate tumorigenesis
Source: Cancer Cell Int. 2021 Dec 2;21:641. doi: 10.1186/s12935-021-02331-x (PMC8641147; doi:10.1186/s12935-021-02331-x)

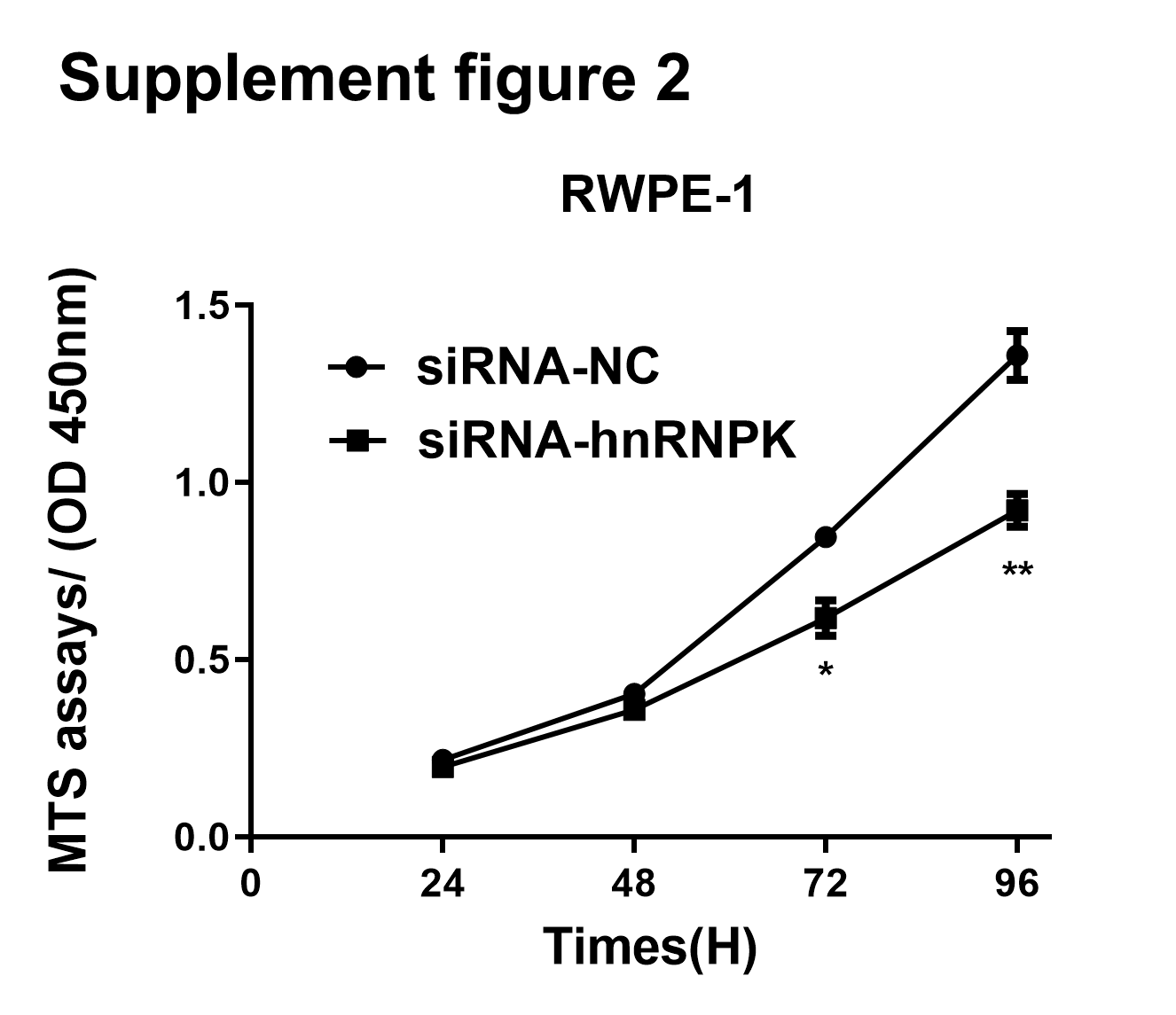

Supplement: Supplementary file 4 — Additional file 4: Fig. S2. Knocked down HnRNPK respectively by using siRNAs in RWPE-1 cells. MTS assays revealed cell viability curves in every 24 h. [file 12935_2021_2331_MOESM4_ESM.tif]

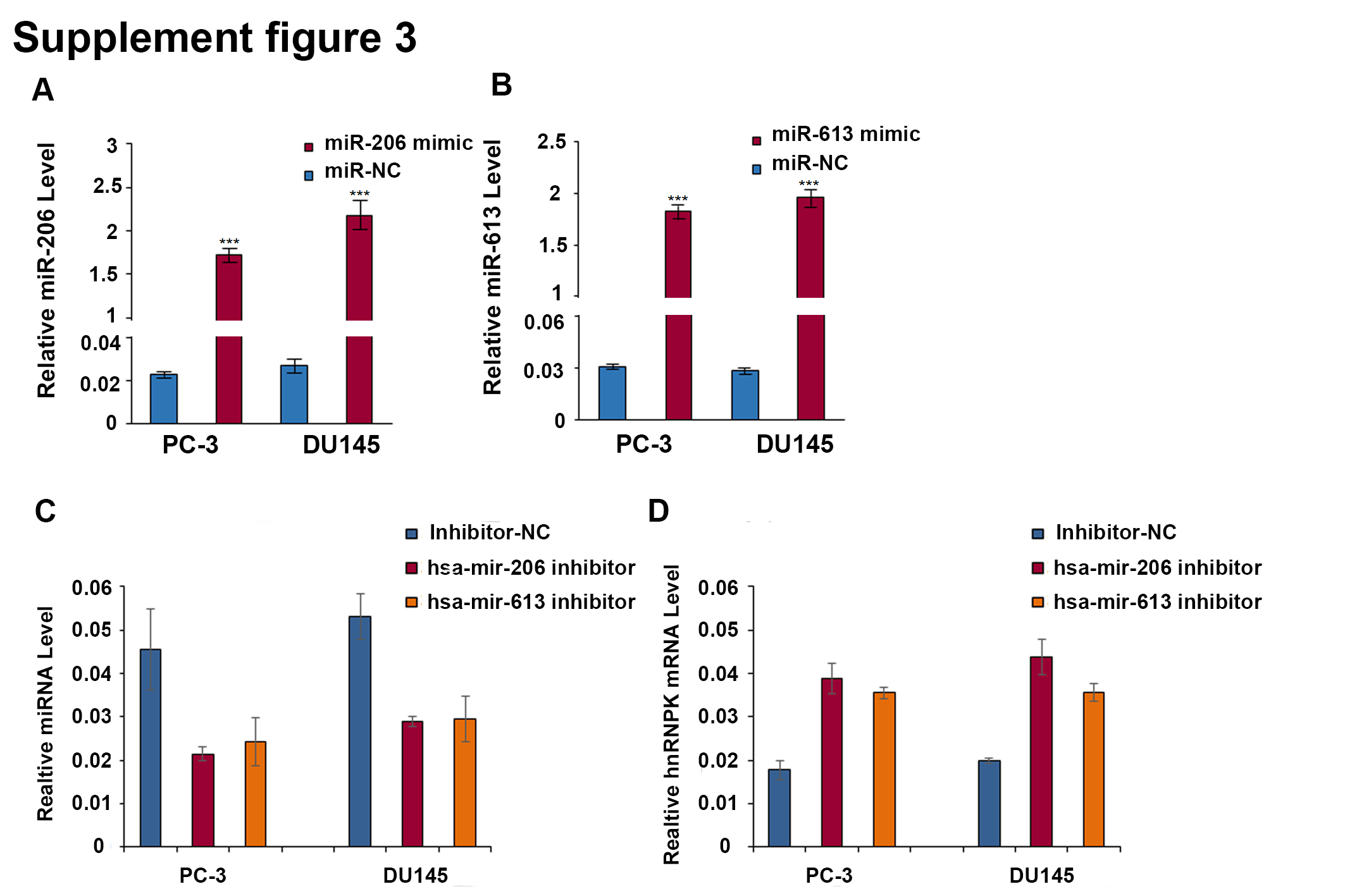

Supplement: Supplementary file 5 — Additional file 5: Fig. S3. A–D Expression of miR-206, miR-613 and HnRNPK in PrCa cells transfected with corresponding miRNA mimics or inhibitor was detected by RT-qPCR and Immunoblot analysis respectively.* P < 0.05; ** P < 0.01; *** P < 0.001 [file 12935_2021_2331_MOESM5_ESM.tif]
